# Machine learning classification of first-episode schizophrenia spectrum disorders and controls using whole brain white matter fractional anisotropy

**DOI:** 10.1186/s12888-018-1678-y

**Published:** 2018-04-10

**Authors:** Pavol Mikolas, Jaroslav Hlinka, Antonin Skoch, Zbynek Pitra, Thomas Frodl, Filip Spaniel, Tomas Hajek

**Affiliations:** 1 Department of Psychiatry and Psychotherapy, Otto von Guericke University, Leipziger Str. 44, 39120 Magdeburg, Germany; 2 3rd Faculty of Medicine, Charles University, Ruska 87, 100 00 Prague, Czech Republic; 3 National Institute of Mental Health, Topolova 748, 250 67 Klecany, Czech Republic; 4 Institute of Computer Science of the Czech Academy of Sciences, Pod Vodarenskou vezi 271/2, 182 07 Prague, Czech Republic; 5 MR Unit, Department of Diagnostic and Interventional Radiology, Institute for Clinical and Experimental Medicine, Videnska 1958/9, 140 21 Prague, Czech Republic; 6 Faculty of Nuclear Sciences and Physical Engineering Czech Technical University in Prague, Prague, Brehova 78/7, 110 00 Praha, Czech Republic; 7 Department of Psychiatry, Dalhousie University, QEII HSC, A.J.Lane Bldg., Room 3093, 5909 Veteran’s Memorial Lane, Halifax, NS B3H 2E2 Canada

**Keywords:** First-episode schizophrenia spectrum disorders, Diffusion tensor imaging, Support vector machines, Magnetic resonance imaging

## Abstract

**Background:**

Early diagnosis of schizophrenia could improve the outcome of the illness. Unlike classical between-group comparisons, machine learning can identify subtle disease patterns on a single subject level, which could help realize the potential of MRI in establishing a psychiatric diagnosis. Machine learning has previously been predominantly tested on gray-matter structural or functional MRI data. In this paper we used a machine learning classifier to differentiate patients with a first episode of schizophrenia-spectrum disorder (FES) from healthy controls using diffusion tensor imaging.

**Methods:**

We applied linear support-vector machine (SVM) and traditional tract based spatial statistics between group analyses to brain fractional anisotropy (FA) data from 77 FES and 77 age and sex matched healthy controls. We also evaluated the effects of medication and symptoms on the SVM classification.

**Results:**

The SVM distinguished between patients and controls with significant accuracy of 62.34% (*p* = 0.005). Participants with FES showed widespread FA reductions relative to controls in a large cluster (N  = 56,647 voxels, corrected *p* = 0.002). The white matter regions, which contributed to the correct identification of participants with FES, overlapped with the regions, which showed lower FA in patients relative to controls. There was no association between the classification performance and medication or symptoms.

**Conclusions:**

Our results provide a proof of concept that SVM might help differentiate FES patients early in the course of illness from healthy controls using white-matter fractional anisotropy. As there was no effect of medications or symptoms, the SVM classification seemed to be based on trait rather than state markers and appeared to capture the lower FA in FES participants relative to controls.

## Background

Neuroimaging has the unique ability to noninvasively investigate brain structure and function. Yet, the diagnostic promise of brain imaging in psychiatry has not been fully realized. Machine learning techniques, which allow for identification of subtle disease patterns on a single subject level, could help realize the diagnostic potential of MRI in psychiatry.

Schizophrenia significantly contributes to the global burden of the diseases [1] and is among the most costly disorders [2]. It is frequently associated with brain imaging alterations, already early in the course of illness. Early diagnosis of schizophrenia may improve prognosis and treatment outcomes [3–5]. Thus, studies applying machine learning to brain imaging data from participants with first episode of schizophrenia-spectrum disorders (FES) are needed.

Previous brain imaging applications of machine learning in schizophrenia have mostly used gray-matter structural or functional MRI data. These studies yielded promising results [6]. Other modalities, such as whole brain diffusion tensor imaging (DTI) have not yet gained comparable attention [7]. Fractional anisotropy (FA) is a widely used DTI measure of diffusivity, which has gained popularity in clinical applications, such as differentiating between ischemic and haemorrhagic stroke [8]. In psychiatry, the FA is used to describe the properties of tissue microstructure [9]. Participants with FES show alterations in microstructural properties of diffuse white matter tracts [10–12].

The previously documented FA differences between FES and control participants, may suggest that FA might be of diagnostic use on an individual level. Yet, there is only a single previous DTI machine learning study [7] in19 pairs of FES and control participants, which reported a 65.79% classification accuracy. More and larger studies are needed to investigate the diagnostic potential of DTI in early stages of schizophrenia [13]. Here, we investigated whether machine learning applied to brain DTI data would differentiate between 77 FES and 77 control participants.

## Materials and methods

### Subjects

This project was a part of ongoing Early Stages of Schizophrenia (ESO) study [10]. The patients with FES were recruited during their first psychiatric hospitalization according to the following inclusion criteria: 1) the diagnosis of schizophrenia, or acute and transient psychotic disorders according to the ICD-10, 2) less than 24 months of untreated psychosis. We excluded patients with psychotic mood disorders, including schizoaffective disorder, bipolar disorder, and unipolar depression with psychotic symptoms. The diagnosis was made by a board certified psychiatrist using the Mini-International Neuropsychiatric Interview [14]. We were primarily interested in individuals at the early stages of illness, in order to limit the effects of medications, co- morbid conditions and previous psychotic episodes. The patients who did not meet the duration criteria for schizophrenia received the working diagnosis of acute and transient psychotic disorders, which is compatible with the brief psychotic disorder according to DSM-IV.

We acquired the MRI scans during the hospitalization, as soon as participants were able to understand and undergo the study procedures. We rated the symptoms at the time of scanning using the Positive and Negative Syndrome Scale (PANSS) [15] and collected information about current treatment. Most patients took medication at the time of scanning, including olanzapine *N* = 29, risperidone *N* = 24, quetiapine *N* = 5, amisulprid *N* = 3, aripiprazole *N* = 4, clozapine N = 2, ziprasidone *N* = 1, haloperidol N = 3, flupenthixol N = 1, medication naive N = 1, n/a *N* = 4.

We recruited the healthy control subjects (HC) through an advertisement from a similar sociodemographic background. We matched the healthy and FES participants individually by age and sex. We applied the following exclusion criteria for the healthy controls: 1) a personal lifetime history of any psychiatric disorder established by the Mini International Neuropsychiatric Interview, 2) family history of a psychiatric illness in first or second degree relatives.

Exclusion criteria common to both groups included: Any current neurological disorders, a lifetime history of seizures, or a head injury, stroke or intracranial haemorrhage, mental retardation, history of substance dependence, and any contraindication of MRI scanning.

### Image acquisition and quality control

We performed the MRI scanning in the Institute of Clinical and Experimental Medicine in Prague on a 3 T Siemens scanner with a Spin-Echo EPI sequence with 2 acquisitions in 30 diffusion gradient directions, TR = 8300 ms, TE = 84 ms, 2 × 2 × 2 mm3 voxel size, b-value 900 s/mm2. DWI data were first visually inspected to check their quality. Subjects with excessive image distortion due to B0 inhomogeneity were excluded. Individual DWI volumes of each subject were inspected and when containing artifacts (k-space spikes, signal void due to movement) were excluded from further processing. If the number of volumes with artifacts per subject was greater than 11, the subject was excluded completely. There was no difference in the mean dislocation parameter between the two groups (t(152) = 0.711, *p* = 0.727).

### Data preprocessing

As described in our previous study [10], we preprocessed the DWI data using FSL tools [16]. Movement and eddy current distortions were corrected by affine registration using FLIRT. The mean dislocation estimated by FLIRT was checked and one subject with excessive value (6.4 mm) was replaced. Maximal value of mean dislocation per subject included in the study was 3.2 mm. The skullstrip was done by BET. The eigenvalues, eigenvectors and subsequent fractional anisotropy, axial and radial diffusivity were estimated by DTIFIT.

To foster compatibility with other studies, we chose an established method of FA preprocessing – the Tract Based Spatial Statistics, implemented in the FMRIB’s Software Library (FSL) [7, 17–22]. We used the standard protocol, as described in the TBSS manual. All of the subjects’ FA data were registered to a pre-defined target FMRIB58_FA using nonlinear registration FNIRT [23]. Next, we created a common skeleton representing all major white matter tracts. As it is necessary to maintain the train/test data separation in machine learning analyses, we did not use the study-specific skeleton option. Instead, we used the standard skeleton derived from the FMRIB58_FA template, as recommended by the manual. The white-matter skeleton was thresholded at recommended 0.2 FA threshold. Finally, all FA data were projected onto this skeleton. As a result, each subject was represented by a single 3D skeletonized FA image.

### Machine learning analyses

We examined the diagnostic utility of the most standard and widely used ML paradigm, the support vector machines (SVM). Specifically, we applied a linear SVM implemented in the PRONTO toolbox v 2.0 to pre-processed skeletonized FA images from 77 FES patients and 77 controls. A common mask was applied to exclude voxels, which were not present in all subjects [24]. The common mask contained 129,154 voxels.

A linear SVM is suitable for analysing high dimensional data such as whole-brain scans while keeping the computational pipeline relatively simple with low computational requirements [25, 26]. This makes it superior for potential clinical setting over complex machine learning pipelines. We used a linear kernel SVM, which is less prone to overfitting than non-linear SVMs. Similar to other studies, we used the default parameter C = 1 [26–28]. The C parameter controls the trade-off between having zero training errors and allowing misclassifications. The performance of the SVM does not change for a large range of C values and only degrades with very small values of C [25]. Modification of the C may be more relevant when the dimensionality of the data is smaller than the sample size [29]. Although a sample-dependent optimization of the C parameter might improve the performance of the model, such approach would contradict our research intentions. Our goal was to reduce the methodological heterogeneity and use a simple, ‘out of the box’ approach potentially applicable in clinical setting [26]. Optimization of the C parameter would introduce methodological heterogeneity, would require nested-cross validation, would make it more difficult to compare the results to other studies and therefore would reduce the potential for a clinical use. Therefore in keeping with other studies and our objectives, we decided to use C = 1.

We used one-to-one matching with regards to age and sex, the most relevant demographic covariates which may affect the FA. The classification itself was performed in a leave-two-out manner. On each run, one patient and one control of the same age and sex were assigned to a testing set. Therefore the classification itself was always performed on participants, who did not differ in relevant demographic variables, but only differed in the presence or absence of psychiatric diagnosis. The resulting cross-validation procedure comprised 77 folds.

We calculated the classification accuracy as the total number of correctly classified test subjects divided by the total number of subjects (154). We tested the statistical significance of the resulting classification accuracy on 1000 randomly permuted datasets, in which all subjects were randomly assigned to a group. The *p*-value of the accuracies was calculated using a resulting null-hypothesis distribution, i.e. as the proportion of the permutations that yielded a greater accuracy than the accuracy found for the classification models.

### Effects of medication and symptoms

We applied 3 different approaches to assess the effect of medication and symptoms. We used an independent-sample t-test to compare the correctly and incorrectly classified subjects. We used the Platt scaling to convert the SVM prediction function values to posterior probability estimates which provide optimal probabilistic interpretation of the SVM output [30] and investigated the association between these estimates and clinical variables. Finally, in order to explore the effects of clinical variables on the FA data itself, we used another machine learning approach – the kernel ridge regression (KR) implemented in PRONTO Toolbox v. 2.0 [24, 31]. KR utilizes multivariate information to predict a continuous variable. We tested whether KR could predict chlorpromazine dose and symptom levels, from the multivariate patterns of FA data.

### Discriminating maps (SVM weight vector)

A weight vector and an offset describe the SVM decision hyperplane. The weight vector corresponds to the most discriminating direction between the groups and is the spatial representation of the decision boundary. We plotted the weight map as a brain image in order to illustrate the relative contribution of the brain regions to differentiation of FES participants from controls.

### TBSS between groups comparisons

In order to indirectly compare the ability of SVM to make prediction about the individual subjects with the actual between-group differences in FA, we compared the skeletonized FA data between FES and HC. This was performed using the Randomise tool [32, 33], with the threshold free cluster enhancement (TFCE) for the family-wise error (FWE) correction at *p* < 0.05 [34]. The regions with significant differences in FA were labelled according to JHU ICBM-DTI-81 White Matter Labels and Tractography Atlas provided within the FSLView package.

## Results

### Demographic data

Our sample consisted of 77 FES patients and 77 age-matched healthy controls without a personal or a family history of psychiatric disorder. For a detailed description of the samples please see Table 1.

**Table 1:** Demographic and clinical data of the healthy controls and the FES participants  in our sample

	Controls (*n* = 77)	FES participants (*n* = 77)	Note
Sex – female N (%)	34 (44%)	34 (44%)	NS
Age, mean (S.D.)	28.32 (7.02)	28.51 (7.03)	*t* = 0.16; *p* = 0.87
Diagnosis (Schizophrenia/Acute polymorphic psychotic disorder) (%)	n/a	46 (59.7%) / 31 (40.3%)	n/a
Median duration of illness, months (SD)	n/a	3 (7.1)^a^	n/a
Drug dose upon MRI – median chlorpromazine equivalent (SD)	n/a	337 (234.8)^a^	n/a
PANSS positive mean (SD)	n/a	13.9 (4.9)	n/a
PANSS negative mean (SD)	n/a	15.7 (6.1)	n/a
PANSS general mean (SD)	n/a	32.8 (8.5)	n/a
PANSS total mean (SD)	n/a	62.4 (16.7)	n/a

*S.D.* Standard Deviation, *MRI* Magnetic Resonance Imaging, *PANSS* Positive and Negative Syndrome Scale

^a^Data from 1 patient missing

### Classification of patients and controls

**Fig. 1:**
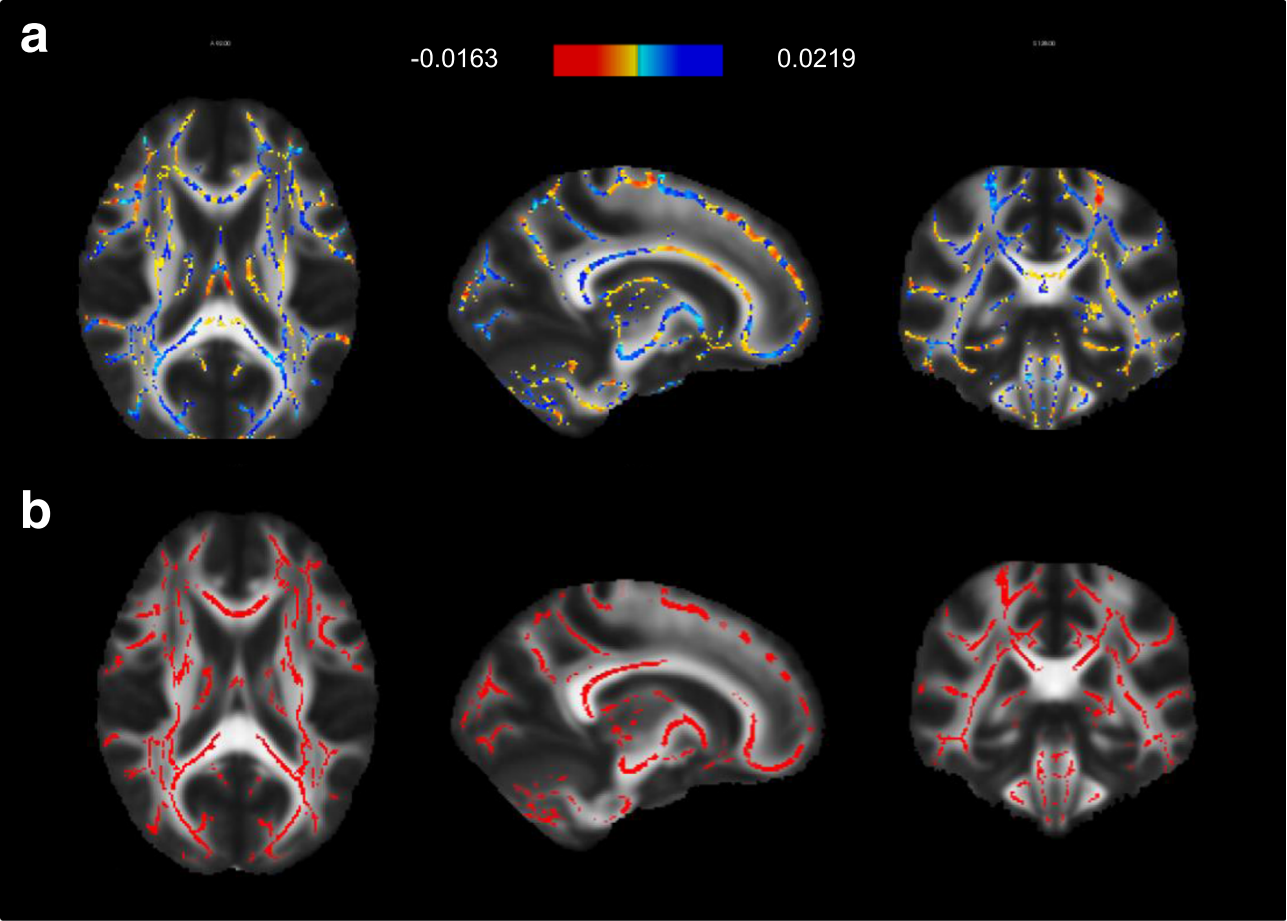
Relative contributions of white-matter regions to the SVM classification and localization of between group differences in FA. **a** SVM weight maps for classification of FES and controls. Maximum weights were diffusely distributed across the main white-matter tracts. **b** Significant FA differences between FES and controls (patients<controls test) (*p* < 0.05 FWE corrected, MNI template). The between group differences in FA overlapped with regions which contributed to classification of FES and control participants on individual level

The SVM classification yielded statistically significant accuracy of 62.34% (*p* = 0.005) and specificity of 64.94% (*p* = 0.005). The sensitivity of 59.74% did not reach statistical significance (*p* = 0.053). In other words, 46 out of 77 patients were correctly classified as cases, whereas 50 out of 77 healthy controls were correctly classified as controls. The anatomical regions with the highest contribution to the differentiation of FES from controls were diffusely spread along the major white matter tracts, see Fig. 1a.

The correctly and incorrectly classified patients did not differ in PANSS score, subscale scores or medication dose on the day of scanning (PANSS Total t(75) = 1.39, *p* = 0.17; PANSS Positive, t(75) = 0.96, *p* = 0.34; PANSS Negative, t(75) = 1.52, *p* = 0.07; PANSS General, t(75) = 1.08, *p* = 0.14; CPZ equivalent t(74) = 0.8, *p* = 0.43). There was no association between the probabilistic estimates of the prediction function value and medication or symptoms CPZ r(74) = 0.09, *p* = 0.42; PANSS Total r(75) = 0.13, *p* = 0.25; PANSS Positive r(75) = 0.072, *p* = 0.54; PANSS Negative r(75) = 0.13, p = 0.25; PANSS General r(75) = 0.12, *p* = 0.29). Kernel ridge regression failed to predict either of the clinical variables from the FA (CPZ r(74) = 0.02, p = 0.29; PANSS Total r(75) = − 0.15, *p* = 0.7; PANSS Positive r(75) = − 0.34, *p* = 0.97, PANSS Negative r(75) = 0.03, *p* = 0.25; PANSS General r(75) = − 0.15, *p* = 0.69).

### Statistical analyses of FA differences

Participants with FES showed widespread FA reductions relative to controls (Fig. 1b). These were contained in a single cluster localized to bilateral tracts of anterior and posterior limbs of the internal capsule, inferior and superior longitudinal fasciculus, inferior fronto-occipital fasciculus, hippocampus, anterior, posterior and superior corona radiata, corpus calossum, cerebral peduncles, inferior, middle cerebellar peduncles and medial lemnisci (size = 56,647 voxels, maximum differences at x = 78, y = 84, z = 32, corrected *p* = 0.002). We identified no areas where FA was significantly greater in patients than controls. The localization of the between-group differences in FA overlapped with the regions, which contributed to differentiation of FES from control participants on the individual level, see Fig. 1a and b.

## Discussion

Machine learning applied to the whole brain FA maps differentiated patients with FES and healthy controls with above chance accuracy of 62.34% (*p* = 0.005). The classification was mostly based on voxels diffusely spread over the major white-matter tracts, rather than localized into specific subregions, see Fig. 1a. The white matter regions, which contributed to the correct identification of participants with FES, overlapped with the regions showing lower FA in patients than controls (Fig. 1b).

Although the accuracy was significantly above chance level, it was relatively low. This could be due to MRI modality, i.e. the use of DTI, or the clinical characteristics, i.e. FES, who may show a lower extent of abnormalities than participants with chronic, long standing illness. We obtained comparable prediction accuracy as the previous study in FES using FA (65.79%) [7]. Previous studies showed greater accuracies between 80 and 90.62% for differentiating participants with chronic schizophrenia from controls [35, 36]. A previous metaanalysis [6] reported accuracies of 80.3% for 38 MRI and/or rsfMRI studies combined. Recent studies in patients with chronic schizophrenia using MRI or fMRI reported prediction accuracies from 85 to 93% [33–35]. Overall, there seems to be a general trend towards higher prediction accuracies in chronic stages of disease, which might reflect the higher prevalence of structural/functional changes due to the disease progression. Although direct comparison is not possible due to methodological heterogeneity, the available studies show that relative to other modalities, DTI gives lower accuracies in both FES and chronic schizophrenia.

Interestingly, we found marked and diffuse differences in FA between FES and controls when using standard methods of between group comparisons. The discrepancy between the large effect size and significance of between-group differences and a relatively low prediction accuracy obtained by machine learning was surprising. It may be related to the type of ML analyses, feature selection, SVM settings, which may affect the sensitivity of the analyses. As our intentions were clinical, we used a simple, standardized approach potentially suitable for clinical application. Having to fine tune the analyses to specific sample would complicate clinical utility. The “out of the box” approach appears to work well for other MRI modalities, such as MRI or fMRI [4, 26]. In our previous study using resting state fMRI data, SVM analyses with default settings were more sensitive than between group comparisons [4]. Perhaps DTI analyses require different default settings or different machine learning algorithms, such as random forest or discriminant analyses. Fine-tuning the SVM models to DTI or finding a ML algorithm better suited for DTI analyses, is beyond the scope of this article, but would be a rich topic for future methodological research.

We replicated previous findings showing that white matter alterations in FES are diffuse and not localized [37, 38]. In keeping with this, the machine learning algorithm used diffuse patterns of white matter changes to identify FES participants. In all instances lower FA values were associated with the diagnosis of FES. Lower FA may indicate that white matter tracts are less organized, have lower density, lower degree of myelination, more crossing fibers or that the membranes are more permeable [9]. Overall, these findings support the growing evidence suggesting disruption of white matter microstructure in FES, which could possibly be used diagnostically.

This study has several limitations. The participants were receiving medications and experiencing moderate symptoms at the time of scanning. Previous studies suggested that there is no association between antipsychotic treatment or symptoms and reduced FA in FES [39]. This is in fact one of the advantages of using DTI relative to fMRI. In keeping with this, we found no effect of medications or symptoms on the classification accuracy.

A common limitation of machine learning studies is overfitting. In order to minimize its risk we used the linear SVM. The relatively low classification accuracy argues against overfitting. SVMs are among the most used ML classifiers in psychiatric neuroimaging [40]. By using SVM we aimed to reduce the methodological heterogeneity and make our analyses better comparable to other studies. For the same reason we used the linear SVM with default parameters. Our results show, that even a relatively simple classifier with linear decision boundary could accurately differentiate patients from controls.

With regards to limitations, in a clinical inpatient setting, where all patients present with marked symptoms, it is more relevant to differentiate between psychiatric diagnoses than between patients and controls. We did not recruit a comparison group of participants with for example first episode of mania. Only few studies have addressed differential diagnosis between major classes of psychosis [41, 42]. More future studies should focus on this important and clinically relevant issue, specifically among participants early in the course of illness.

## Conclusions

In summary, this is a proof of concept that machine learning applied to the whole brain FA values may help differentiate FES from healthy controls on an individual level, even when using a relatively simple machine learning classifier. As there was no effect of medications or symptoms, the SVM classification is likely based on trait rather than state markers. Given the marked differences in FA between FES and controls obtained by the TBSS analyses the classification accuracy was relatively low. Due to this discrepancy, it is possible that a different ML algorithm might improve the classification accuracy.

## Abbreviations

CPZ: Chlorpromazine equivalent
DTI: Diffusion tensor imaging
FA: Fractional anisotropy
FES: First episode of schizophrenia-spectrum disorder
HC: Healthy control subjects
KR: Kernel ridge regression
ML: Machine learning
MRI: Magnetic Resonance Imaging
PANSS: Positive and negative syndrome scale
rsfMRI: Resting-state functional MRI
SVM: Support-vector machines
TBSS: Tract based spatial statistics
TFCE: Threshold free cluster enhancement
WM: White matter
